# Blastomycosis Misdiagnosed as Tuberculosis, India

**DOI:** 10.3201/eid2509.190587

**Published:** 2019-09

**Authors:** Anil Kumar, Akhilesh Kunoor, Malini Eapen, Pradeep Kumar Singh, Anuradha Chowdhary

**Affiliations:** Amrita Institute of Medical Sciences and Research Centre, Kochi, India (A. Kumar, A. Kunoor, M. Eapen);; Vallabhbhai Patel Chest Institute, Delhi, India (P.K. Singh, A. Chowdhary)

**Keywords:** *Blastomyces dermatitidis*, tuberculosis, TB, granuloma, blastomycosis, fungi, India, tuberculosis and other mycobacteria

## Abstract

Chronic pulmonary blastomycosis is often misdiagnosed and treated as tuberculosis in disease-endemic and non–disease-endemic areas. We report the case of a 32-year-old man who after visiting Chicago, Illinois, USA, returned to India and received treatment for tuberculosis for 12 months before receiving the correct diagnosis of blastomycosis.

*Blastomyces dermatitidis* is a dimorphic fungus that is rarely reported from India; no well-defined area of endemicity in that part of the world has been recorded (*1*). However, it is endemic to the Ohio and Mississippi River valleys of North America and the states bordering the Great Lakes (*2*). Acute pulmonary infection is caused by inhalation of aerosolized *B. dermatitidis* conidia, which convert to yeast forms within the lungs (*3*). In the acute stage, blastomycosis may be misdiagnosed as bacterial pneumonia and sometimes as another illness. Most cases of blastomycosis are usually diagnosed after the infection has become chronic. Severe pulmonary disease can occur in apparently immunocompetent, as well as immunocompromised, persons (*3*). Persons from non–disease-endemic areas usually acquire this disease during travel to disease-endemic areas (*1*).

In November 2014, a 32-year-old man, native to the state of Kerala, India, sought care for multiple discharging sinuses on his anterior chest wall (Figure, panel A). He weighed 75 kg and had been receiving first-line antituberculosis (anti-TB) therapy for 12 months for nonresolving left upper lung lobe consolidation. Skin on his legs and forearms showed patchy hair loss without erythema, nodularity, or scarring. Past records showed that sputum, bronchoalveolar lavage fluid, and pus from a cold abscess were negative for *Mycobacterium tuberculosis* by smear, culture (BACTEC MGIT 960; Becton Dickinson, https://www.bd.com), and PCR (Cepheid Xpert MTB-RIF, http://www.cepheid.com). A fine-needle aspirate, obtained from the left upper lung lobe with computed tomography guidance, showed suppurative granulomas. Results of Mantoux, anti–cyclic citrullinated peptide, antinuclear antibody testing, and serologic testing for HIV were negative. In the absence of a definitive diagnosis, first-line anti-TB therapy was empirically initiated and continued for another 12 months without any clinical improvement.

**Figure:**
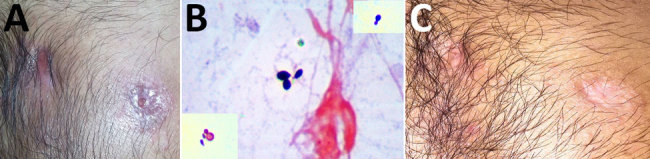
Patient with blastomycosis, India, 2014. A) Photograph of chest showing actively discharging sinuses before treatment with antifungal medication. B) Slide of Gram-stained pus discharge, showing broad-based budding yeast cells. The insets show Gram staining of the same organism, with narrow and broad-based budding in different fields. Original magnification ×100. C) Photograph of chest showing closed sinuses and disappearance of sinus line after treatment with antifungal medication.

High-resolution computed tomography of the chest showed consolidation with air space opacities, and multiple subcutaneous pockets of pus with discharging sinuses above the sternum were noted. Detailed travel history revealed that before the illness, the patient had worked on a 9-month project in Chicago, Illinois, USA, during which time he resided in Lisle, Illinois. He did not indulge in outdoor activities that may increase the possibility of inhaling spores of this fungus, such as river rafting or hiking.

At the Department of Microbiology, Amrita Institute of Medical Sciences and Research Centre, Kochi, India, Gram and calcofluor white staining of the pus collected from the discharging sinus showed budding yeast cells (Figure, panel B). Examination of a pus smear revealed no acid-fast bacilli. Pus was cultured on Sabouraud dextrose agar and 5% sheep blood agar. Biopsy samples from lesions on the forearm, stained with periodic acid–Schiff and Grocott-Gomori methenamine silver, were negative for fungal elements. 

Because of strong suspicion of a fungal infection, probably blastomycosis, and considering the patient’s stay in Chicago, anti-TB therapy was replaced with itraconazole at a dose of 200 mg 2×/d. Cultures on Sabouraud dextrose agar grew a dimorphic fungus identified microscopically as *B. dermatitidis* and confirmed by sequencing (GenBank accession no. KT443881). Antifungal susceptibility (according to the Clinical and Laboratory Standards Institute, https://clsi.org) showed low MICs for itraconazole (0.06 μg/mL), voriconazole (0.25 μg/mL), amphotericin B (0.5 μg/mL), micafungin (0.125 μg/mL), anidulafungin (0.06 μg/mL), and caspofungin (0.25 μg/mL). After 12 months of antifungal therapy, the chest wall sinuses closed and the sinus lines disappeared (Figure, panel C). High-resolution computed tomography showed complete healing of left upper lobe lesions, which had resulted in focal fibrosis and cystic and tubular traction bronchiectasis. At this time, antifungal therapy was discontinued.

Chronic pulmonary blastomycosis results in chronic cough, weight loss, and hemoptysis, often masquerading as TB or malignancy (*4*). The patient described here exemplifies the challenges of diagnosing pulmonary blastomycosis in a non–blastomycosis-endemic area where TB is prevalent. Most patients receive multiple courses of anti-TB treatment, which can delay blastomycosis diagnosis by >1 month (*5*). Chronic pulmonary blastomycosis has been misdiagnosed and treated as TB in disease-endemic and non–disease-endemic areas (*1*,*4*). Even in blastomycosis-endemic areas such as Illinois, the median time from onset to diagnosis is 128 days (range 12–489 days) (*6*). The presence of skin lesions increases the recognition of blastomycosis (*2*). The patient reported here had worked for 9 months in an area where *B. dermatitidis* is highly endemic (*6*). Presence of skin lesions, negative mycobacterial cultures and Xpert MTB/RIF assay results, and the absence of response to anti-TB treatment should have raised the suspicion of blastomycosis for this patient.

Definitive diagnosis of blastomycosis can be made only by culture, which often takes weeks. Direct potassium hydroxide smears and cytopathology are inexpensive, produce rapid results, and can demonstrate characteristic broad-based budding yeasts in samples (*1*). Although the sensitivity of urinary antigen test for blastomycosis is high, that test lacks specificity because of cross-reactions with *Histoplasma* spp. (*7*). 

Blastomycosis is rarely reported in India; a review by Kumar et al. (*1*) reported only 6 definitively diagnosed cases, of which 2 were associated with travel to disease-endemic areas in the United States (*8*,*9*). The choice of antifungal medication for blastomycosis depends on disease severity. For severe disease, the recommended treatment is initial amphotericin B therapy for 1–2 weeks followed by oral itraconazole; for mild and moderate disease, the recommended treatment is oral itraconazole. A minimum of 6 months of treatment is required for all patients with pulmonary blastomycosis (*8*).

A high index of suspicion is needed to detect blastomycosis in non–disease-endemic areas where TB is prevalent. Clinicians should elicit a thorough travel history from patients with illness that does not respond to anti-TB treatment.
